# Professionals’ perceptions of interprofessional collaboration within condition-based units

**DOI:** 10.1371/journal.pone.0343792

**Published:** 2026-06-08

**Authors:** Sara M. de Jong-Ultee, Liselot de Waard, Dorine J. van Staalduinen, Machteld J. Mosselman, Dennis van Veghel, Lea M. Dijksman, The St. Antonius condition-based unit working group, Paul B. van der Nat

**Affiliations:** 1 Department of Value-Based Healthcare, St. Antonius Hospital, Nieuwegein/Utrecht, The Netherlands; 2 Scientific Center for Quality of Healthcare (IQ Health), Radboud University Medical Center, Nijmegen, The Netherlands; 3 Department of Obstetrics, OLVG hospital, Amsterdam, The Netherlands; 4 The scientific institute, Martini Hospital, Groningen, The Netherlands; 5 Santeon, Utrecht, The Netherlands; 6 Heart center, Catharina Hospital, Eindhoven, The Netherlands; Public Library of Science, UNITED KINGDOM OF GREAT BRITAIN AND NORTHERN IRELAND

## Abstract

**Introduction:**

Hospitals can form condition-based units (CBUs) to support value-based healthcare (VBHC). Interprofessional collaboration is key to the success of CBUs, but insights into its quality are lacking. We aimed to examine professionals’ perceived quality of interprofessional collaboration in CBUs, and factors influencing it.

**Methods:**

A single-center mixed-methods study was conducted within four CBUs in a Dutch top-clinical hospital: (1) geriatric trauma, (2) prostate cancer, (3) colon cancer and (4) breast cancer. A relational coordination (RC) survey was used to examine professionals’ perceived collaboration quality, followed by semi-structured interviews to explore influencing factors.

**Results:**

Varying RC scores were observed between professionals with different professions and disciplines within CBUs (geriatric trauma: 1.9–5.0; prostate cancer: 2.9–4.8; colon cancer: 2.1–4.8; breast cancer: 1.1–4.9), with higher scores among professionals involved in similar parts of related care pathways. Interview findings suggest that this variation is explained by four factors: (1) streamlined communication, (2) active engagement, (3) mutual recognition and (4) workflow efficacy.

**Conclusion:**

This exploratory study introduces a novel approach to studying professionals’ perceived collaboration quality in CBUs through relational dynamics. The results suggest that traditional hospital structures can hinder interprofessional collaboration in CBUs by limiting physical interactions and shared administrative systems. Future studies with larger samples are needed to confirm these findings, and to provide recommendations for improving interprofessional collaboration in CBUs in order to unlock their potential in improving patient care.

## Introduction

Value-based healthcare (VBHC) is an organizational strategy that can be considered as a global trend in healthcare management [1]. It focuses on rewarding providers based on patient health outcomes instead of the number of health services delivered [2]. A key element of implementing VBHC is organizing care around conditions, which requires collaboration between groups from different professions (e.g., doctors and nurses) and disciplines (e.g., oncology and pathology) to provide the full care pathway [3]. To organize around conditions, hospitals aiming to implement VBHC therefore started implementing condition-based units (CBUs) in their organization. The current VBHC literature proposes four types of condition-based organizations (CBOs) that hospitals may use to structure their care delivery around conditions [4]:

1. **Multidisciplinary project teams**, where the structure of traditional functional departments is the primary structure. CBUs in the organization are project teams formed by different professional groups, focused on value improvement for specific medical conditions, with limited formal responsibilities.
2. **Matrix organization**, where, in addition to the structure of functional departments, there are formal CBUs and reporting lines around care delivery for specific conditions.
3. **Integrated Practice Units (IPUs)**, where the structure of CBUs is the dominant organizational framework.
4. **Independent Treatment Center (ITC)**, an independently operating healthcare organization focused on the complete care cycle for a specific medical condition.

The success of the implementation of VBHC, in any of the four types of CBOs, is suggested to depend on the quality of interprofessional collaboration between professional groups [3]. As a concrete example, in an IPU aimed at delivering care for stroke patients, individuals with various professions (e.g., physical therapists focused on the recovery phase and nutritionists offering support services) collaborate across various disciplines to address patients’ needs [5]. Working within an IPU includes interprofessional meetings to discuss cases, as well as working together on continuous value improvement.

Previous research suggests that interprofessional collaboration in CBOs can be challenging [6]. Not every professional group has sufficient opportunities to collaborate with others. For example, in a matrix organization, certain disciplines, such as professionals from the pathology departments, must allocate their time across multiple medical conditions within functional departments, which can limit their time for the CBU. Furthermore, professional groups with diverse work activities may find it challenging to connect with each other due to differing perceptions of these activities [7,8]. For example, nurses and physicians may differ in their norms and values about their interprofessional work. Nurses are traditionally trained in interprofessional collaboration and shared decision-making with physicians, and believe their perspectives should be valued in decision-making. In contrast, physicians, trained on technical skills and focused on a specific specialty, are more accustomed to practicing independently. As a result, nurses see physician-nurse collaboration as more critical for patient care compared to doctors. These professional groups may also have limited understanding of each other’s roles and responsibilities [9]. For instance, physicians may view nurses solely as responsible for implementing treatment orders rather than contributing to decision-making [8]. This perception can diminish nurses’ sense of autonomy and professional worth in decision-making processes.

The importance of interprofessional collaboration around conditions to the success of VBHC, alongside the challenges professionals can encounter when working interprofessionally, imply that we must focus on better understanding and improving the quality of this collaboration. However, detailed insights into the quality of interprofessional collaboration in CBUs, including factors that influence it, are lacking. To date, interprofessional collaboration around conditions has only been studied at the level of broad professional groups [9], such as physicians and nurses, but not at the level of their affiliated disciplines (e.g., pathology) and work activities (e.g., treatment orders and treatment decisions). As illustrated above, challenges to the collaboration may also emerge at these levels. Explaining these challenges could provide specific insights into how the collaboration in CBUs can be improved. Therefore, the aim of this study was to investigate perceptions of professional groups – characterized by professions and disciplines – about interprofessional collaboration within CBUs and factors influencing this collaboration.

The quality of interprofessional collaboration in CBUs can be investigated using the concept of relational coordination (RC) [10,11], which has been previously applied in a study on IPUs [9]. RC can be used to examine team dynamics, and in particular, the quality of communication and relationships among members of interprofessional teams. According to RC literature, high quality collaboration is characterized by 1) relationships of shared goals, knowledge, and mutual respect and 2) communication that is frequent, timely, accurate and problem-solving. These two dimensions – communication and relationships – mutually reinforce each other, thereby enabling team members to effectively coordinate the work process in which they are engaged together.

This study focuses on the interprofessional collaboration between professional groups within CBUs using the concept of RC, also considering the various work activities in which they are engaged. As such, it recognizes that the quality of interprofessional collaboration may vary across the care pathway due to differing perceptions of the collaboration among groups involved. The insights generated may be valuable for hospital managers in enhancing interprofessional collaboration at the level of CBUs, or specific stages of the care pathway.

## Methods

### Study design

A single-center mixed-methods design was employed. Initially, a validated RC survey was distributed to assess the perceived quality of interprofessional collaboration among professional groups in care chains. Subsequently, in-depth interviews were conducted to better understand the survey results and gain insights into factors influencing the quality of the collaboration.

### Study setting

This study was performed in CBUs established in 2019 at the St. Antonius hospital, a Dutch top-clinical hospital with a matrix organization. These CBUs, referred to locally as ‘care chains’, consist of healthcare professionals with different professions and disciplines who remain part of their pre-existing traditional departments and are governed by a daily board (DB) comprising a medical leader, a nurse leader, and a manager [12].

The study focused on four care chains: 1) geriatric trauma, 2) prostate cancer, 3) colon cancer and 4) breast cancer. The four care pathways these care chains focus on are extensive, involving various professionals collaborating in different compositions. To study the quality of interprofessional collaboration in relevant parts of the care pathways, segments requiring intensive collaboration between different professional groups – characterized by different professions (e.g., physiotherapists and nurses) and disciplines (e.g., radiologists and oncologists) – were selected. This segment was selected for every care pathway with the care chains’ DBs, and visualized by outlining work activities and professional groups.

The Medical Research Ethics Committee United (MEC-U) affirmed that the study does not fall within the Medical Research Involving Human Subjects Act (reference number W23.109). Therefore, ethical approval was not required. Prior to participation, informed consent was ensured from participants through written communication along with verbal communication. They were made aware of their right to withdraw from the study at any time.

### Sampling

The selection of eligible participants was led by the expertise of the care chains’ DBs using purposive sampling to ensure diverse perspectives. All healthcare professionals working in the CBUs were considered eligible, though they had to collaborate with at least two other professional groups within the CBU. The DBs selected professional groups intensively collaborating with other groups in the care pathway segments. For each professional group, they provided the research team with a list of names of individuals collaborating with other professional groups in the care chain. This approach ensured that all eligible participants, other than the clinical champions, were invited to participate in the study.

The research team e-mailed eligible participants from the four care chains an online survey regarding the interprofessional collaboration in their care chain through REDCap (Research Electronic Data Capture) between May 11 and June 19, 2023. They received weekly reminders for a total duration of four weeks to encourage timely responses.

At the end of the survey, participants were asked if they would be willing to be interviewed to provide additional insights. Those who expressed interest were invited for the interviews, which took place from June 5 to October 31, 2023.

### Data collection

#### Survey.

The perceived quality of interprofessional collaboration was evaluated using questions from the Dutch translation of Gittell’s RC survey [13]. This survey involves Likert-scale responses from 1.00 (never) to 5.00 (always) to statements on each of the seven RC elements.

#### Interviews.

At the start of each interview, participants were asked to offer feedback on visualizations of the care pathway segments. This was done to verify the segments’ completeness. Subsequently, open-ended questions were asked to identify factors influencing the quality of interprofessional collaboration (S1 Table). For instance, participants were asked what they consider essential for effective collaboration.

### Data analysis

#### Survey.

Average RC scores were computed from survey responses by averaging the seven RC elements in IBM SPSS version 25. Scores from questionnaire responses with more than three missing items for a professional group were excluded. Average RC scores were descriptively analyzed – in the context of the care pathway segments – to assess the perceived quality of interprofessional collaboration. The minimal dataset of this study has been uploaded as S1 Data.

#### Interviews.

Interviews were audiotaped, transcribed and analyzed through thematic analysis in ATLAS.ti using an inductive approach for coding [14]. To ensure the reliability, interviews were double-coded. Interview data from the geriatric trauma and prostate cancer care chains were analyzed by the first author (SdJU) and the second author (LW). Furthermore, interview data from the breast cancer and colon cancer care chain were analyzed by the first author (SdJU) and the fourth author (MM).

During the interview analyses the authors, who both engaged in multiple qualitative education courses prior to the study, independently assigned codes to specific text segments in the transcripts. Inter-coder discussions were conducted after 20%, 60%, and 100% of the interview analyses to address any discrepancies in coding. All disagreements were resolved before proceeding with further analysis. In case consensus could not be achieved between the two coders, a third author (DS) was consulted to provide adjudication. Data saturation was achieved when no new codes emerged and agreement continued between the coders, indicating that all relevant themes had occurred. After reaching consensus on the codes, they were organized into thematic categories, which were subsequently discussed within the research team (S2 Table).

## Results

119 Eligible professionals (Geriatric Trauma: 40, Prostate Cancer: 22, Colon cancer: 23, Breast cancer: 34) received the online survey. In total, 60 professionals (response rate 50.4%) completed the survey. Survey data from 59 professionals (49.6%) was included in the analysis (Table 1). 20 Interviews were performed.

**Table 1:** Professional groups of survey and interview participants (N = 59, N = 20).

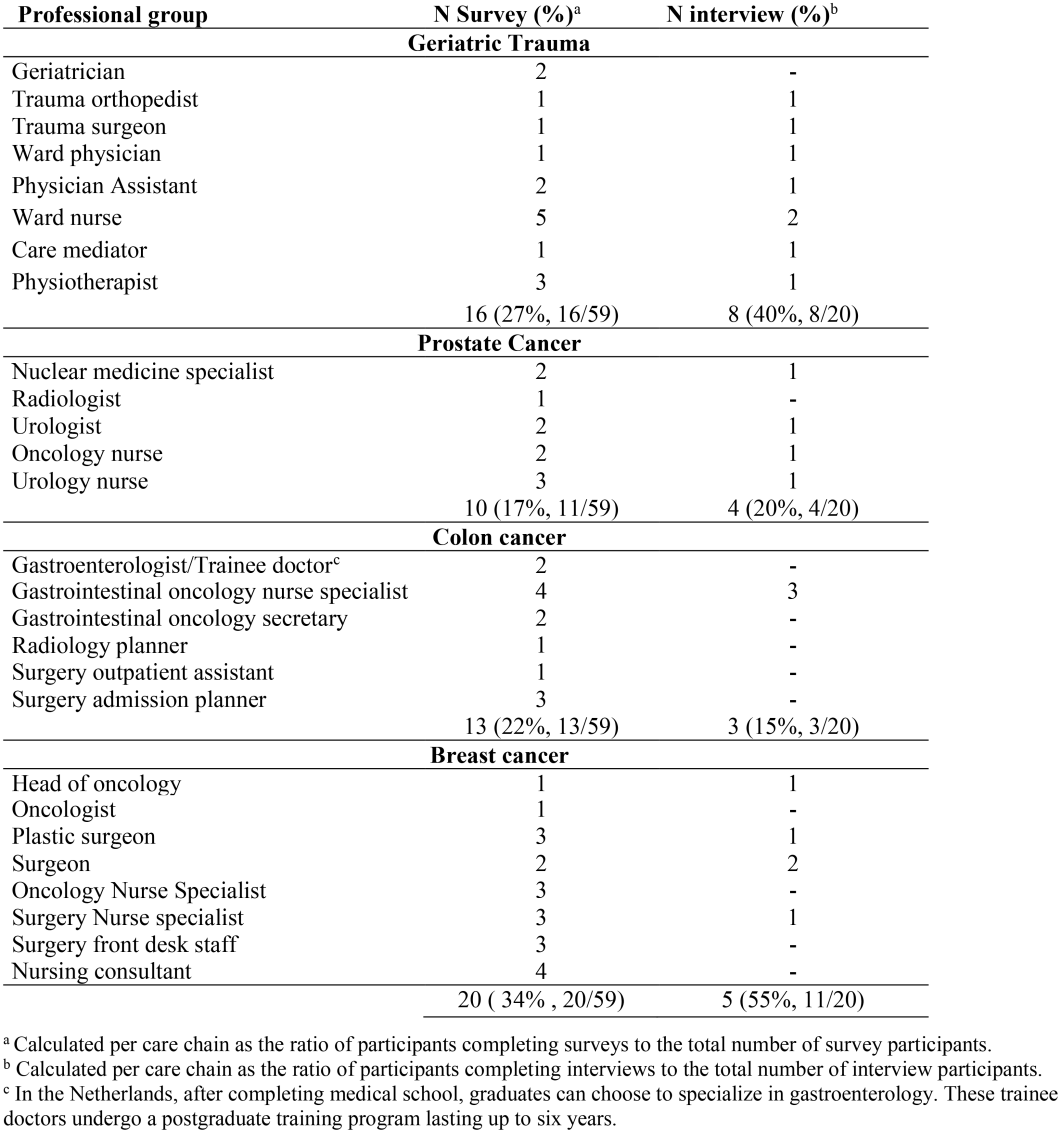

### Geriatric trauma care chain

The selected care pathway segment for the geriatric trauma care chain starts with the patient’s admission to the ward for medical examinations, medical policy determination, and pre-operative screening if surgery is needed (Table 2). After surgery, the patient returns to the ward for postoperative care.

**Table 2:** The studied geriatric trauma care chain segment, including professional groups selected to participate.

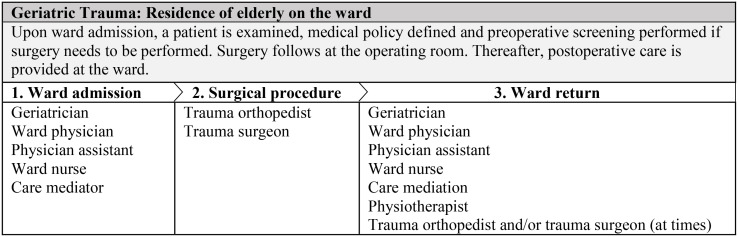

#### Quantitative results.

Relatively higher RC scores were observed among professionals active at the nursing ward (3.3–5.0), and average scores among medical specialists active at the surgical procedure (3.3–3.4) (Table 3). Relatively higher scores were also observed for the nursing ward professionals as rated by medical specialists (2.8–5.0). In contrast, relatively lower RC scores were observed for medical specialists as rated by nursing ward professionals (ward nurses (2.4–3.1), care mediators (2.6), and geriatricians (1.9–2.6)).

**Table 3:** Average RC scores in the Residence of elderly on the ward.

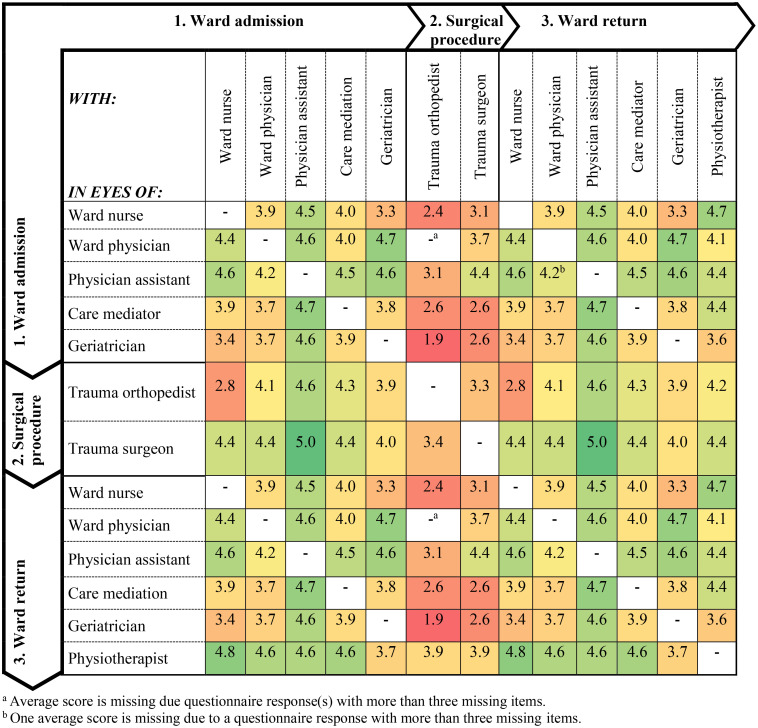

#### Qualitative results.

The interviews revealed that the quality of interprofessional collaboration was perceived to be lower under circumstances of limited opportunities for being engaged in the collaboration in the geriatric trauma care chain. One professional active at the nursing ward expressed that it is difficult to approach some medical specialists involved in surgical procedures who are physically less present.

“I feel more hierarchy in orthopedics than in surgery. […] Trauma surgeons are approachable and willing to explain their choices. And trauma orthopedists, well, you hardly ever see them.” (R1)

Interestingly, beyond physical presence, professionals expressed that their sense of being able to engage with others is shaped by opportunities they receive and seize to be included. For example, a medical specialist active at the surgical procedure, expressed that his discipline was not engaged enough by everyone in the care chain, and experienced limited capacity for the care chain.

“I think that the care chain leader sees it as a kind of one-man show instead of really picking it up together with other healthcare providers, like the trauma orthopedists.“ (R8)

“There are time constraints because a colleague who is also specialized in traumatology […] left to another clinic. Now, I have to do everything by myself’‘ (R8)

In contrast to what was described about engaging with professionals active at the surgical procedure, nursing ward professionals expressed having sufficient opportunities for meaningful engagement with each other. They described a pro-active approach in anticipating to each other’s needs, which reduces the workload for others and stimulates a mutually established division of tasks in the collaboration.

“If we know that some patients will have lunch in the living room at the end of the hallway, a few of us will walk with the patients to the living room. This way, they receive extra supervised mobilization, and the nurses do not have to walk back and forth to take them to the living room. In this way, we work in mutual cooperation.’‘ (R6)

“After I finish my morning rounds and approach the PA with my questions, she assures me she’ll handle it. I know she’ll get back to me later that day. Then I often ask her if there is anything I need to do, and we quickly divide the tasks between us.’‘ (R1)

In addition to opportunities for engagement, nursing ward professionals also highlighted their appreciation of each other’s contributions to decision-making.

“Twice a week we have a multidisciplinary meeting [with the geriatrician, physiotherapist, and care mediation. [..] Important decisions are made. I think you shouldn’t make these alone.” (R3)

“I appreciate being able to make decisions together with the geriatrician about whether to continue treatment. A while ago, I had a case with the geriatrician. We had been actively treating a patient for a week, but there was no improvement. Eventually, we had a conversation with the family about whether to continue treatment or transition to comfort care.“ (R3)

In addition to the collaboration among professionals active at the nursing ward, the interviews also revealed a positively experienced collaboration among professionals active at the surgical procedure. Professionals from both groups expressed the importance of physical proximity for these collaborations. This allows nursing ward professionals to easily reach out to each other for urgent patient cases, and medical specialists to ask each other questions outside of formal meetings.

“We have three regular physician assistants. If I’m concerned about a patient, I just have to enter their room and say, ‘Come with me now.’ […] They have a small office in the middle of the ward.” (R1)

“We can easily run to geriatricians on the ward if issues with a patient arise. We have had similar positive experiences with ward physicians.“ (R5)

“Good communication is the kind of contact you have in the hallway: being able to reach out to each other when you have a question. […] We do not have structural meetings with [these medical specialists]. We see each other in the hallway’‘ (R8)

### Prostate cancer care chain

For the prostate cancer care chain, the segment’s first activity is the doctor’s referral in the triage, upon which the diagnostic pathway is determined (Table 4). If diagnostic tests confirm the diagnosis of prostate cancer, a treatment plan is formulated during the weekly multidisciplinary team meeting (MDTM).

**Table 4:** The studied prostate cancer care chain segment, including professional groups selected to participate.

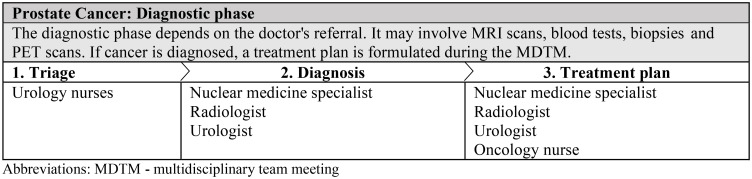

#### Quantitative results.

Relatively higher scores were observed among medical specialists involved in the diagnosis and treatment plan formulation (3.6–4.8) and relatively higher scores were observed for nurses involved in the triage and treatment plan formulation as rated by these specialists (3.9–4.8) (Table 5). In contrast, relatively lower RC scores were observed for these medical specialists as rated by these nurses (2.9–3.1).

**Table 5:** Average RC scores in the Diagnostic phase.

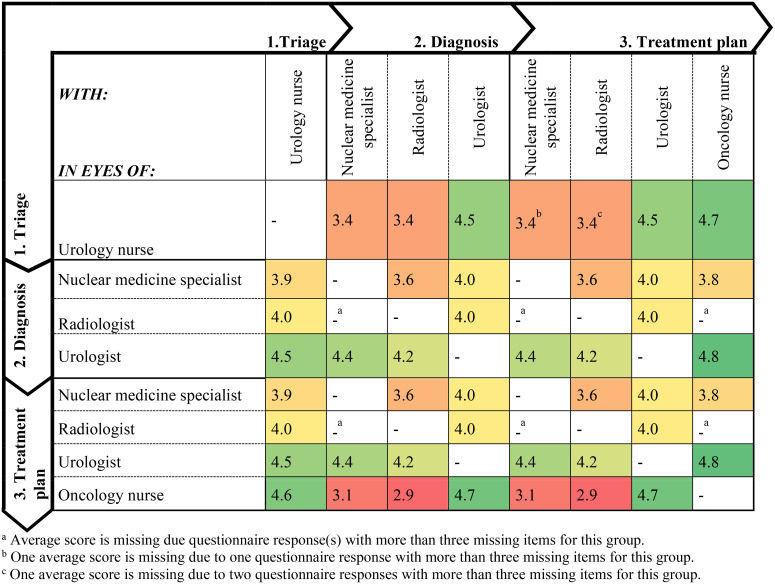

#### Qualitative results.

Respondents indicated that seeing each other at the workplace is important for staying engaged together in the collaboration within the prostate cancer care chain. An example is one’s presence at interprofessional meetings. A professional involved in the treatment plan formulation mentioned that engaging with medical specialists, involved in the diagnosis and treatment plan formulation, would improve if they would be present at these meetings.

“It would be nice if individuals [you want to approach] are present at lunch meetings […]. For example, radiologists […], we never see them there. […] If you are a care chain together, you should probably ensure that there is someone or something from each discipline present.” (R13)

Similarly, one of these medical specialists expressed feelings of being overlooked by other professional groups in the care chain. This seems to make it difficult to engage with others in the collaboration.

“The urology department is the main driving force. […] At times, we feel overlooked, which is unfortunate. […] Collaboration with colleagues could be improved.’‘ (R11)

The finding that seeing each other may improve professionals’ perceived interprofessional collaboration within the prostate cancer care chain is further supported by a positively expressed collaboration with urologists, and among medical specialists. One professional described being able to easily reach out to urologists at the workplace, who seem to be more involved than other medical specialists. Furthermore, another professional described that frequent physical interactions among medical specialists facilitate informal contact.

“Throughout the day we have contact with the urologist […] Very often, it’s just a quick phone call. Or when I’m present at their work I just pop in.’‘ (R12)

“[…] Urologists are super involved and motivated. They see each other at coffee breaks, so they interact a lot. [Other medical specialists] are further away, just like us.” (R11)

“The nuclear medicine specialists, radiologists […] and urologists form a stable team [who see each other regularly at the MDTM]. I am pleased to participate in person, as this provides the opportunity to discuss matters in the hallway before or after the formal meeting.’‘ (R11)

### Colon cancer care chain

In the colon cancer care chain, a colonoscopy is performed after referral for investigation (Table 6). In case of abnormalities, follow-up examinations are scheduled. Thereafter, the patient is discussed in the MDTM, a treatment plan is formulated and surgery is planned.

**Table 6:** The studied colon cancer care chain segment, including professional groups selected to participate.

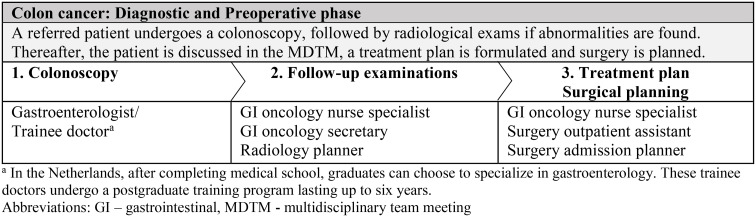

#### Quantitative results.

In the colon cancer care chain, relatively higher scores were observed for other professional groups as rated by the gastroenterologist/trainee doctor, involved in the colonoscopy (Table 7). In contrast, relatively lower scores (2.1–2.3) were observed for the radiology planner, involved in follow-up examinations, as rated by administrative personnel, involved in treatment and surgical planning.

**Table 7:** Average RC scores in the diagnostic and preoperative phase.

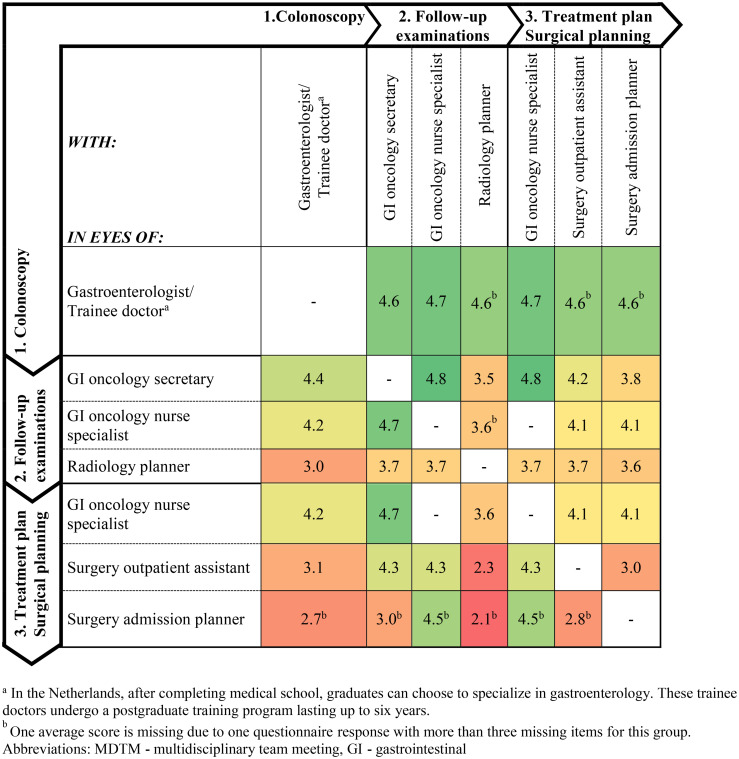

#### Qualitative results.

Several nurse specialists described that their perceived quality of interprofessional collaboration in the colon cancer care pathway is limited by delays in the task of a group of medical specialists. Their work is negatively affected by these delays, as their tasks are then subsequently delayed.

“I see limiting factors for good collaboration in [their] outpatient clinics running late. If [they are] running late, my clinic gets delayed too.’‘ (R18)

“The delay in [their] outpatient clinic is really something unpleasant. […] Our outpatient clinics then also run behind schedule, which causes frustration.’‘ (R17)

A nurse specialist also indicated that coordination issues with this professional group of medical specialists limit the perceived quality of interprofessional collaboration. Alternating contact of various nurse specialists with patients creates uncertainty for this group about who is responsible for the patient.

“If I have spoken to a patient and I receive a message from [this medical specialist] while my colleague has spoken the patient afterwards, it can be inconvenient.“ (R16)

One nurse specialist indicated that the perceived quality of collaboration with medical specialist is enhanced by their availability for communication. This seems to enable them to provide high-quality patient care together.

“We can help patients in an early stadium [because we] can easily ask a surgeon, ‘Can you take a look?’ or a radiologist, ‘Could you check that scan in advance?’” (R17)

### Breast cancer care chain

In the breast cancer care chain, the segment starts with patients’ referral for investigation, after which diagnostic examinations are scheduled (Table 8). If examinations confirm breast cancer, a treatment plan is formulated in the MDTM, and treatment is started afterwards.

**Table 8:** The studied breast cancer care chain segment, including professional groups selected to participate.

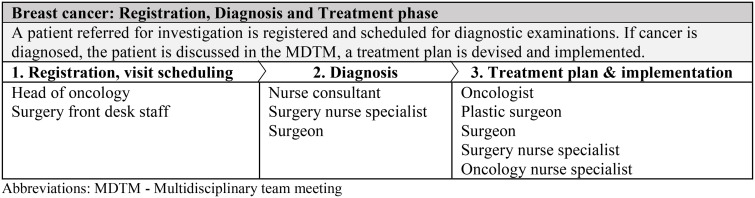

#### Quantitative results.

In the breast cancer care chain relatively higher scores (3.5–4.7) were observed for the surgeon and surgical nurse specialists as rated by all other disciplines, with the exception of the department head of oncology involved in managing front desk staff (Table 9). This department head was also observed to assign relatively low scores to all other disciplines (1.1–3.0), and to receive lower scores from the surgical nurse specialist (1.4) and nurse consultants (1.4) – involved in the diagnostic and/or treatment phase.

**Table 9:** Average RC scores in the registration, diagnosis and treatment phase.

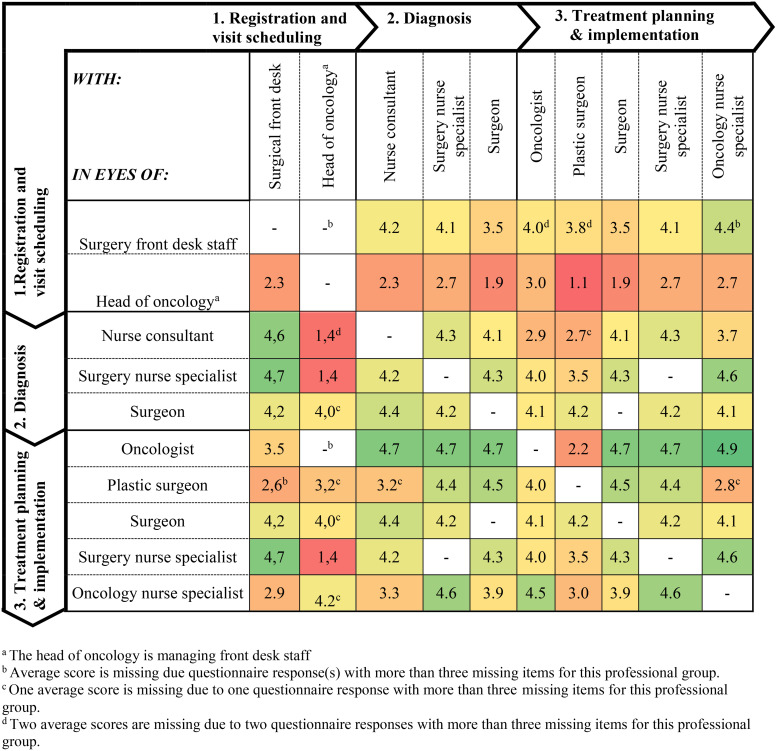

#### Qualitative results.

Professionals in the breast cancer care chain indicated that the complex structure of this care chain can hinder opportunities for collaboration. For example, there are two administrative teams with their own departmental rules and scheduling systems. The department head described that it has been challenging to let these teams collaborate because they tended to stay within their own silos.

“It is complex here. We have our administrative team [internal medicine], and next to us, there is the administrative team of the breast center. […] They have their own departmental rules.’‘ (R20)

“It has been very difficult to let [these two teams] collaborate with each other. [Both teams] were in their own tunnel, used to working in their own way. They also physically sat apart from each other […].’‘ (R20)

Furthermore, the different scheduling systems are not accessible to everyone. A nurse specialist described this makes it difficult to coordinate logistics and determine who will be treating a particular patient.

“We cannot access each other’s systems. Let’s say the patient [wants a specific caretaker to look at his/her complaints]. I can never see when [he/she] is available because the surgical appointment schedule and oncological appointment schedule do not align.’‘ (R19)

Professionals indicated that physical proximity at the workplace positively influences their perceived quality of interprofessional collaboration in the breast cancer care chain. For example, one medical specialist described that running clinics at the same site and time with another professional group, enables asking each other questions or discuss uncertainties.

“We always run a clinic at the same time as [nurse specialists], so they can always ask us questions, and we can also ask them questions.’‘ (R15)

Furthermore, other medical specialists reported that working in the same physical space with another group enhances insights into each other’s work. This seems to enable better coordination of tasks, leading to improved patient care.

“We frequently perform surgeries with the plastic surgeon. Therefore, we can always ask questions or discuss uncertainties with them during a surgical day’‘ (R14)

“Some surgeons say, ‘Oh, let person A perform that surgery, because he/she does it more often.’ […] Then you provide better care each time. […] But then you have to know how others carry out their work.” (R21)

Professionals also highlighted in the interviews that expressing appreciation for each other’s work positively influences the perceived collaboration in the breast cancer care chain. At the workplace, medical specialists often give informal feedback to their colleagues, highlighting what they appreciate about their work. This seems to help creating a work environment where people feel recognized.

“At Christmas, we distribute Dutch pastries in our funny Christmas sweaters at the departments. Then, we write down what we appreciate about the departments on a card. In every department, I visit the coffee room where I share the positive feedback we hear from patients and observe ourselves. And that is greatly appreciated.“ (R14)

### Overarching factors explaining relational coordination

In the interviews four overarching factors emerge that are suggested to contribute to the variation in RC scores among diverse professional groups within care chains. These factors reflect how the interviewed professionals perceive the quality of collaborating with other professional groups in care chains. First, these professionals described streamlined communication, facilitated by physical proximity and accessibility, as supporting interactions. Second, professionals reported that opportunities to engage together in the care chain – stimulated by open, active, and inclusive behavior – increase their opportunities to approach each other. Third, they suggested that an efficient workflow eases task coordination. Fourth, they described that mutual recognition, which involves appreciating and acknowledging each other’s skills, contributes to an inclusive and positive work environment.

## Discussion

To examine professionals’ perceptions of the quality of interprofessional collaboration in CBUs and the factors influencing it, this study employs a single-center mixed-methods design – combining a RC survey with interviews – across four CBUs. The observed variation in RC scores suggests variation in the perceived quality of interprofessional collaboration between professional groups – characterized by different professions and disciplines – within the CBUs in this study. In general, the perceived quality of interprofessional collaboration seems to be higher among professional groups involved in similar parts of CBUs’ care pathway segments (e.g., triage, diagnosis, treatment planning) and lower among those involved in different parts. The interviews suggest that this variation in perceptions may be explained by four factors: (1) streamlined communication, (2) active engagement, (3) efficacy of the workflow and (4) mutual recognition. We discuss this link below by relating the study’s quantitative findings – where higher RC scores indicate a stronger, and lower RC scores a weaker, perceived quality of collaboration – to qualitative results presenting explanatory factors.

First, in this single-center study we observed that the perceived quality of interprofessional collaboration in the CBUs, measured with RC, relates to the level of streamlined communication. Streamlined communication, in this context, refers to being able to easily interact with each other, for example through physical proximity or accessibility for communication. The level of streamlined communication within CBUs seems to be complicated by traditional function-based hospital structures. Some professions seem to be traditionally more co-located together than others. For example, in the geriatric trauma care chain in this study, a functional divide exists between professionals active at the nursing ward and medical specialists who are rarely present at the nursing ward. As a result, the quality of interprofessional collaboration, reflected by RC scores, appears to be stronger within these groups of ward professionals and medical specialists – where informal and frequent interactions are more easily facilitated – than between the groups. This finding relates to Porter’s description of success ingredients for IPUs. He emphasizes that realizing physical proximity between professionals by collocating them in an appropriate physical setting improves collaboration for it allows them “to easily coordinate, see patients together and interact formally and informally on a daily basis [3, p. 10]’‘. This link is supported by research on interprofessional collaboration in healthcare demonstrating that physical proximity fosters regular interactions between professionals [15]. Our study results suggest, and therefore we hypothesize, that within the CBU context, this positive relationship between physical proximity and the level of interprofessional collaboration can be attributed to physical proximity’s impact on facilitating more streamlined communication.

Second, in the studied hospital we observed that the quality of interprofessional collaboration in CBUs depends on opportunities for professionals to engage collectively within CBUs. This involves professionals both having and creating opportunities for shared engagement within the CBU. This study’s results suggests that when professionals have and create opportunities to engage with one another, they build stronger relationships and foster more effective communication – reflected in higher RC – which ultimately enhances their perceived quality of collaboration. Conversely, the results propose that medical specialists who are less visible at the workplace – and therefore more challenging to engage with – tend to receive lower RC scores. The traditional functional hospital structure may explain why this latter relationship seems to occur quite frequently. A previous study showed that it is difficult for some disciplines to be engaged in CBUs because they have to split their time between various conditions, which stems from the traditional hospital structure [6]. Nevertheless, not all medical specialists in this study seem to suffer from these limitations for they appear highly motivated and engaged, and receive high RC scores. We suggest that this relates to their intrinsic motivation to collectively engage with other professionals in the CBU. This motivation for active engagement with others could stem from a strong sense of belonging to an interprofessional team [16]. Therefore, the high engagement of certain medical specialists in this study could indicate a strong identification with the CBU. However, further quantitative and qualitative research is needed to understand how this sense of belonging relates to the opportunities individuals create for engaging with each other in the CBU.

Third, our observations within the studied organization seem to reflect that workflow inefficiencies negatively influence the way professionals perceive the quality of interprofessional collaboration in CBUs. These inefficiencies involve, for example, working with multiple scheduling systems, which cannot be accessed by everyone and therefore complicate smooth collaboration among professionals. In particular, the findings suggest how workflow inefficiencies in CBUs may hinder task coordination among CBU professionals, and therefore negatively influence professionals’ perceptions on the quality of their collaboration. Similar to the factors described above, these issues seem to relate to the traditional functional hospital structure. Often, the workflow and involved administrative systems remain designed according to traditional specialty-based departments. This can be problematic for the CBUs for it can hinder professionals linked to different departments to coordinate their tasks needed for the efficient delivery of patient care [17]. This signifies not only the importance of a single scheduling structure, an important feature of IPUs [3], but also of an organizational structure that facilitates and supports coordination between professional groups in CBUs.

Fourth, our observations suggest that mutual recognition may positively influence the perceived quality of interprofessional collaboration among professionals in CBUs. Mutual recognition in CBUs involves acknowledging each other’s work by considering each other’s input in interprofessional meetings and expressing signs of appreciation for each other’s work. This study’s results indicate that professional groups who express recognition for the work of other groups in the CBU experience a stronger interprofessional collaboration with these individuals, as reflected by the higher RC scores between these groups. Recognizing each other in interprofessional teams requires a willingness to respect and understand each other’s work [18]. This may be especially relevant in CBUs, where respect for others’ work is crucial due to the high interdependencies among professionals. One of the key elements for effectively coordinating interdependent team work is relationships of mutual respect [19]. Respect encourages team members to value each other’s contributions, to consider the impact of their actions on others and to consider the needs of the entire team. Furthermore, better understanding of each other’s work seems important in CBUs, as the various roles and responsibilities of professionals are not always clear. For example, there can be confusion regarding the decisional mandate of the CBU’s management and its relation to the mandate of traditional department managers [6].

Our mixed-methods study using the concept of RC in CBUs enhances the understanding of interprofessional collaboration within a VBHC context. It also introduces a novel approach to evaluating the quality of collaboration in CBUs by analyzing relational dynamics. However, this approach is not easily applicable in daily hospital practice. The methodology – RC surveys and semi-structured interviews – requires significant time. Completing the survey takes approximately 20 minutes per individual across professional groups, while interviews require an additional 45 minutes per person. Although this approach presents valuable insights, applying theories on relational dynamics, such as RC, for continuous evaluation and performance improvement in hospitals necessitates further research. Future efforts should focus on developing and testing a practical RC-based tool to measure collaboration quality continuously in CBUs.

### Implications for practice

The findings of this single-center study suggest that working in physical proximity enhances opportunities for professional groups to collectively engage with one another, and eventually improves interprofessional interactions and informal communication. While these findings do not demonstrate causal effects, they may indicate that opportunities for professional groups to work in shared physical work environments can facilitate interprofessional collaboration. This may be achieved by supporting multidisciplinary activities requiring the work of different professional groups, strongly encouraging presence at interprofessional team meetings and facilitating shared workspaces for professionals within CBUs.

Second, the findings of this study seem to imply that some professionals, especially those who are understaffed or have demanding schedules due to responsibilities beyond CBU-related tasks, may experience difficulties engaging sufficiently in CBUs due to limited capacity. This highlights the potential relevance of professionals’ transparency about capacity constraints, as well as managers’ efforts to increase opportunities for professionals’ engagement, although further research is needed to explore these dynamics more systematically.

Third, this study seems to suggest that different administrative teams with separate rules and scheduling systems can cause coordination issues in CBUs. While these findings should be interpreted cautiously, they may point towards the potential relevance of more frequent interactions among administrative personnel in CBUs to support a more unified way of working, as well as shared access to the scheduling systems for professionals.

Finally, beyond these broad and tentative implications for practice, this study also seems to provide context-specific insights into the quality of interprofessional collaboration within the four CBUs. Therefore, the approach used in this study may serve as a reflective tool that CBUs can use to identify areas for improvement and to improve interprofessional collaboration within their specific context.

### Limitations

Several limitations may have influenced our findings. First, most of our findings may reflect context-bound patterns. Exceptions to these patterns are our findings regarding physical proximity, which are supported by studies in other healthcare settings [9,15], including hospitals with IPUs [9]. Our study was conducted exclusively in CBUs of one hospital with a matrix organization. Because the traditional function-based structure and the newer condition-based structure in this organization are equally important [4], professionals in CBUs must continue to use administrative systems linked to the traditional structure and divide their time between the focal condition in the condition-based structure and other conditions in the traditional structure which limits interprofessional collaboration quality. Furthermore, the collaboration quality may be limited by hierarchical differences among healthcare professionals in the traditional structure, as previous studies have shown that such differences can negatively affect the quality of team-based work [20]. These issues may be less pronounced in CBOs, where the traditional function-based structures are less prominent. Therefore, future studies are encouraged to replicate this study across hospitals with various forms of CBO in order to enhance the external validity of our findings.

Second, a notable limitation of this study is the sampling method, which relied on a list provided by the care chain’s DBs. Although this approach was necessary to ensure participants were involved in intensive collaboration within the selected segment of the care pathways, it may have introduced selection bias, as it favored the most engaged professionals within the care chains. While this choice offered comprehensive insights into the factors shaping collaboration within CBUs, the focus on these actively engaged professionals may have limited our understanding of factors influencing the collaboration involving less actively engaged professionals. Future studies could apply a less strict selection procedure, approaching all involved professional groups.

Third, for the quantitative descriptive part of this study, we identified participants’ function and role within the CBU, and their perceived quality of RC, but we did not examine how long participants occupy their current role in the CBU. Previous research revealed positive relations between RC and healthcare providers’ length of time working in the same team [21], and between RC and regular interprofessional activities [19]. These relations imply that interprofessional collaboration is facilitated by the time working together. Future research testing our proposed hypotheses should consider including participants’ tenure within the CBU as an additional variable.

Fourth, our sample consisted of a small number of individuals per professional group. This restricts the generalizability of our findings. Furthermore, it may have restricted insights into factors explaining collaboration quality, as not all participants who completed the survey took part in the interviews. Therefore our findings should be interpreted with caution. Consequently, the current study should be viewed exploratory, providing a foundation for future studies. Future studies on interprofessional collaboration in CBUs may solve the issue of small sample sizes by increasing the number of participants through the inclusion of broader professional groups, such as medical specialists and nurses with a specific discipline.

Fifth, we collected self-reported data, which is prone to social desirability bias or recall bias. Therefore, our findings may have been affected if participants over-reported positive behaviors and under-reported negative behaviors. However, the risk of socially desirable responses was reduced because the surveys asked participants to report on the behaviors of others rather than their own [10]. Moreover, because the interviewer was unfamiliar with the participants and their identities remained anonymous, participants were likely more comfortable providing honest and accurate information.

## Conclusion

This exploratory study uses a novel approach to studying collaboration quality in CBUs through relational dynamics. While the findings are preliminary due to the small sample size, they suggest that interprofessional collaboration in CBUs can be especially challenging for professionals involved in different parts of the CBUs’ related care pathways. The observed collaboration challenges seem to arise from issues related to the traditional functional hospital structure in which the CBUs operate. Specifically, limited physical interactions and limited opportunities to work collaboratively, such as with similar scheduling systems, may constrain effective coordination and communication. Physical proximity and similar administrative systems may be especially important in CBUs, as professionals in the CBUs who are linked to different departments may already feel distant from each other due to differences in work locations, focus areas and administrative systems. These findings highlight potential mechanisms to encourage these professionals to collaborate closely around a condition and to foster mutual understanding and recognition of each other’s expertise to deliver high-quality care together. Future studies with larger samples are needed to confirm our findings and to increase the potential of CBUs to improve patient care through high quality interprofessional collaboration in practice.

## Supporting information

S1 Table Interview topic guide. (DOCX)

S2 Table Thematic analysis. (DOCX)

S1 Data Minimal dataset used in the study. (XLSX)
