# Supplementary material for: Professionals’ perceptions of interprofessional collaboration within condition-based units
Source: PLoS One. 2026 Jun 8;21(6):e0343792. doi: 10.1371/journal.pone.0343792 (PMC13245800; doi:10.1371/journal.pone.0343792)
Supplement: S1 Table — (DOCX) [file pone.0343792.s001.docx]

## **S1 Table. Interview topic guide.**

1. Does this outline of the care pathway correspond with your experiences?
2. Is it correct that these individuals are involved in these phases?
3. Could you elaborate on your role in the chain
   - What is your role?
   - Where do you stand in the process?
   - With which of these professions from this list do you mainly interact during your activities?
4. What is essential for you for effective collaboration?
5. Could you provide an example of successful collaboration? Can you give an example of less successful collaboration?
6. What are the barriers you have experienced in collaboration?
7. Does communication with the individuals you collaborate with occur physically, over the phone, or digitally? Would you like to experience it differently?
