# Supplementary material for: Professionals’ perceptions of interprofessional collaboration within condition-based units
Source: PLoS One. 2026 Jun 8;21(6):e0343792. doi: 10.1371/journal.pone.0343792 (PMC13245800; doi:10.1371/journal.pone.0343792)
Supplement: S2 Table — (DOCX) [file pone.0343792.s002.docx]

# **S2 Table. Thematic analysis.**

| **Thematic analysis** | | |
| --- | --- | --- |
| **Code** | **Sub-theme** | **Theme** |
| Knowledge of each other’s work | Reaching the right person in time | Streamlined communication |
| Timeliness |  |  |
| Capacity |  |  |
| Accessibility |  |  |
| Presence at the workplace |  |  |
| Consistency |  |  |
| Regular contact | Meeting structure |  |
| Organization of meetings |  |  |
| Approachability | Open and active attitude | Opportunity to engage |
| Pro-active attitude |  |  |
| Thinking along |  |  |
| Involvement of others | Inclusivity |  |
| Integration of disciplines |  |  |
| Avoid repetition | Task delay and repetition | Efficacy of the workflow |
| Avoid delays |  |  |
| Task integration | Workflow integration |  |
| Task coordination |  |  |
| Acknowledgement of interdisciplinary collaboration | Appreciation of others | Mutual recognition |
| Appreciative environment |  |  |
| Shared goals | Group cohesion |  |
| Trust |  |  |
